# Attitudes toward COVID-19 vaccines during pregnancy and breastfeeding

**DOI:** 10.3389/fpubh.2024.1286891

**Published:** 2024-03-01

**Authors:** Nesibe Simsekoglu, Enes Akyuz, Rabia Guven, Ozge Pasin

**Affiliations:** ^1^Department of Home Patient Care, Hamidiye Vocational School of Health Services, University of Health Sciences, Istanbul, Turkey; ^2^Department of Biophysics, Faculty of International Medicine, University of Health Sciences, Istanbul, Turkey; ^3^Division of Obstetrics and Gynaecology, Beykoz State Hospital, Istanbul, Turkey; ^4^Department of Biostatistics, Faculty of Medicine, Bezmialem University, Istanbul, Turkey

**Keywords:** anti-vaccine movement, attitude to health, breastfeeding, COVID-19 vaccines, pregnancy

## Abstract

**Background:**

Although vaccination is one of the most effective means of controlling the spread of COVID-19, public concerns and indecision about vaccination still continue. Because pregnant and breastfeeding individuals are at high risk for severe outcomes in case of infections, determining their level of hesitation and attitude toward COVID-19 vaccines will guide the management of the disease. This study aimed to determine pregnant and breastfeeding women’s levels of hesitation and attitude toward COVID-19 vaccines as well as their related factors.

**Methods:**

The sample of this descriptive research consisted of 103 pregnant or breastfeeding individuals who were seen at the obstetrics and gynecology outpatients clinic of a state hospital in Istanbul, Turkey. The data were collected using a ‘demographic data form’, the ‘Vaccine Hesitancy Scale in Pandemic’, and the ‘Attitudes toward COVID-19 Vaccine Scale’. The research data were analyzed with appropriate statistical methods.

**Results:**

The mean age of the participants was 29.71 ± 4.75, 51% were pregnant, and 74.8% had received the COVID-19 vaccine. The mean score of the ‘Vaccination Hesitancy Scale in Pandemic’ was 30.83 ± 6.91, and the mean score for the ‘Attitude Scale toward the COVID-19 Vaccine’ was 25.50 ± 5.20. A significant difference was found between the total score of the ‘Vaccine Hesitation Scale in the Pandemic’ and the mean score of the ‘Lack of Confidence’ sub-dimension between the ‘working status’ and the ‘influenza vaccination’ status. In terms of the mean score of the ‘Risk’ sub-dimension, a significant difference was found between the ‘period of vaccination’ (*p* < 0.05). According to the mean total score of the ‘Attitude Towards COVID-19 Vaccine Scale’, there was a significant difference between the ‘smoking’ status. There was a significant difference in the ‘Positive Attitude’ sub-dimension in terms of the ‘flu vaccination’ status. There was a significant difference in the ‘Negative Attitude’ sub-dimension in terms of the ‘chronic disease’ status. A positive correlation was found between the total scores of the scales.

**Conclusion:**

It was concluded that although the participants had a high level of hesitation toward the COVID-19 vaccine, they had a positive attitude. The results obtained will be guided in determining the strategies to be developed for these specific groups in future pandemics.

## 1 Introduction

Coronavirus Disease 2019 (COVID-19), a new infectious disease of the acute respiratory tract has become a global public health problem. In addition to the direct effects of the disease, many indirect factors, including the lack of access to health services and socioeconomic status, negatively affect public health (1–3). According to the World Health Organization (WHO), more than 774 million confirmed cases and more than 7 million deaths have been reported worldwide as of January 2024. Approximately 14 billion doses of COVID-19 vaccine have been administered (4).

During pregnancy or breastfeeding, individuals are at high risk of severe outcomes in case of COVID-19, as they are more susceptible to infectious diseases due to their weakened immune systems. COVID-19 progresses with a clinical picture similar to that of general population, such as colds and flu, in individuals during pregnancy and breastfeeding. However, it has been reported that individuals during pregnancy and breastfeeding are more susceptible to COVID-19 in cases where pregnancy complications occur or chronic diseases are accompanied. Especially in the first trimester of pregnancy, there is a risk of congenital anomalies and miscarriage due to hyperthermia. In addition, risks such as preterm birth, pre-eclampsia, premature rupture of membranes, and the possibility of cesarean section due to fetal distress can be seen in pregnant women who develop pneumonia (3, 5–7).

One of the most important steps to hinder COVID-19 is to prevent transmission. Effective interventions to prevent transmission include: early detection of the disease through nasopharyngeal swabs and serological tests, social distancing, use of medical masks, personal hygiene and vaccination (8–10). Vaccination, especially in the early period of the epidemic, is of great importance for the health of the whole community, especially the older adults and individuals with chronic diseases, who are disadvantaged in terms of infectious diseases (1, 11, 12).

Vaccination during pregnancy is important for the mother’s active immunity and the newborn’s passive immunity against serious infectious diseases (2). Immunoglobulin G (IgG) antibodies are produced by the mother and transferred to the fetus via the placental circulation. Mucosal IgG, IgA and IgM antibodies secreted in colostrum and milk protect the newborn after birth (6, 13, 14). There is not yet enough data on the effects of COVID-19 vaccines on breastfed infants. However, according to a center for Disease Control and Prevention report, the benefits of breastfeeding to the infant and what is known as the safety of other vaccines while breastfeeding, the recently vaccinated individual should be encouraged to start or continue breastfeeding (6, 15, 16).

Safe and effective vaccines are available to protect people from COVID-19 and help terminate the pandemic. However, the success of the vaccine depends not only on its efficacy, but also on its acceptance by the community. It is recommended that approximately 70% of society be vaccinated to ensure effective social immunity against COVID-19. However, in a study centered in Turkey, it was reported that approximately one in three people were hesitant about COVID-19 vaccines (17–19). Vaccine hesitancy is defined as “delay or refusal to accept the vaccine despite the availability of vaccination services” (20). WHO has reported that vaccine hesitancy is one of the top 10 threats to global health. In this context, understanding how individuals perceive the epidemic and their attitudes toward the control of the epidemic will be guiding health professionals in managing the epidemic as well as vaccine supply (18, 20–23).

There are various behavioral and social factors that affect the decision-making and implementation process of individuals regarding vaccination. These factors include vaccine type, geography, culture and socio-economic status (24, 25). The limited data on COVID-19 vaccines and the infodemic lead to some understandable hesitations in pregnant and breastfeeding individuals (26). The literature emphasizes that vaccine-related reactions in pregnant and breastfeeding individuals are similar to the general population. In addition, there are also research results reporting that vaccinated pregnant individuals are less likely to contract COVID-19 and that the disease has a milder course in infants born to vaccinated mothers (3, 5, 7, 13). Nevertheless, lack of trust in healthcare staff is also a crucial factor among the ones that negatively affect vaccination preference. Healthcare professionals are mostly criticized for ignoring patients’ concerns about COVID-19 vaccines. This situation undermines trust in both health professionals and the health system. On the other hand, some health personnel also report that they are not adequately prepared for their role in promoting and providing immunization (24, 27). These results emphasize the importance of the educational role of all health personnel, especially nurses and physicians, in providing and promoting immunization. It is recommended that nurses, who are the primary source of information for pregnant and breastfeeding individuals, provide effective care in the light of evidence-based information by accessing up-to-date information and guidelines on COVID-19 vaccines (14, 27).

Determining the level of hesitancy and attitudes of individuals during pregnancy and breastfeeding toward COVID-19 vaccination and the factors affecting them will guide health professionals in terms of controlling the disease in the early period by increasing vaccination rates in practice and planning protective health policies against future epidemics. This study planned in this direction aims to determine the hesitancy and attitude levels of individuals during pregnancy and breastfeeding toward COVID-19 vaccines and to examine the related factors.

Within the scope of the aim of the study, the following questions were sought to be answered;

What is the level of hesitation of the participants toward COVID-19 vaccines?

What is the level of attitude of the participants toward COVID-19 vaccines?

What are the demographic variables affecting the participants’ level of hesitation and attitude toward COVID-19 vaccines?

Is there a relationship between the level of hesitation and attitude of the participants toward COVID-19 vaccines?

## 2 Methods

### 2.1 Research design

This research was designed as a descriptive cross-sectional type.

### 2.2 Place and time of research

The study was conducted between April and June 2022 in the obstetrics and gynecology outpatients clinic of a public hospital in Istanbul.

### 2.3 Population and sample of the research

The population of the study consisted of individuals in pregnancy and breastfeeding period who applied to the obstetrics and gynecology outpatients clinic of a state hospital in Istanbul. An adequate sample size for the study was calculated using the G-power program. According to this, while the Type I error amount was 0.05 and the test power was 0.80, it was determined that a total of 100 individuals should be reached, 50 during pregnancy and 50 in the breastfeeding period. The study was completed with 103 participants who signed the informed consent form. After the study was completed, *post-hoc* power analysis was performed to determine the adequacy of the sample size. According to the power analysis, it was determined that the total sample was sufficient with an effect size of 1.09, 99.8% power and 0.05% margin of error (28).

### 2.4 Data collection

A questionnaire to be used to collect research data consists of 3 main parts. In the first part, ‘Demographic Data Form’, in the second part, ‘Vaccine Hesitancy Scale in Pandemics (VHSP)’, and in the third part, ‘Attitudes Towards COVID-19 Vaccine Scale (ATCVS)’ are included. Data were collected by face-to-face interview method. It took approximately 7 min to fill out the data collection forms.

#### 2.4.1 Introductory information form

The Introductory Information Form, which was created by the researchers in line with the literature and submitted to the expert opinion, consists of 21 questions in total (20, 29, 30). Ten of the multiple-choice questions in the form assessed the demographic characteristics of the participants, while 11 questions assessed their habits such as smoking, disease and vaccination-related characteristics.

#### 2.4.2 Vaccine hesitancy scale in pandemics

The scale was developed by Larson et al. in 2015. Its Turkish validity and reliability was conducted by Capar and Cınar in 2021. The 5-point Likert-type scale consists of a total of 10 items and two sub-dimensions. Scoring in the scale is: Strongly disagree = 1, Disagree = 2, Neither agree nor disagree = 3, Agree = 4, Strongly agree = 5. While the score to be obtained from the scale varies between 10 and 50, an increase in the total score of the scale indicates an increase in the level of hesitation toward vaccines administered during the pandemic period.

The first sub-dimension “Lack of Confidence” consists of 8 items (M1-T, M2-T, M3-T, M4-T, M5-T, M6-T, M7-T, M8). Inverse items with the letter “T” next to it 1 → 5; 2 → 4; 3 → 3; 4 → 2; It is encoded as 5 → 1. High scores in this sub-dimension indicate that mistrust toward the vaccine increases in pandemics. The second sub-dimension “Risks” consists of 2 items (M9, M10). High scores in this sub-dimension indicate a high risk of vaccination in pandemics. Two factors of the scale explain 68.53% of the total variance. The factor loads for the items were 0.638 and 28, and the Cronbach alpha reliability coefficient was 0.901. In this study, the reliability coefficient of the Vaccine Hesitancy in Pandemics Scale was found to be 0.831 (31).

#### 2.4.3 Attitudes toward COVID-19 vaccine scale

The scale, which was developed by Wide et al. in Turkey in 2020, is a 5-point Likert type and consists of two sub-dimensions (Positive and Negative Attitudes) and 9 items. Expressions on the scale are: Strongly disagree = 1, Disagree = 2, Undecided = 3, Agree = 4, Strongly agree = 5. The attitude score is obtained by summing the scores obtained from the questions in the sub-dimension of the scale and divided by the number of questions. The first sub-dimension, ‘Positive Attitude’, consists of four items (M1, M2, M3, M4). High scores from the ‘Positive Attitude’ sub-dimension indicate that the attitude toward the vaccine is positive. The second sub-dimension, ‘Negative Attitude’, consists of five items (M5-T, M6-T, M7-T, M8-T, M9-T). Items with the letter “T” next to them are reverse items. It is calculated after the items in the ‘Negative Attitude’ sub-dimension are reversed, and the higher scores in this sub-dimension and the total score indicate that the negative attitude toward the vaccine is less. In the study of Wide et al., the Cronbach alpha coefficient of the Attitudes Toward COVID-19 Vaccine Scale was found to be 0.96 for the positive dimension and 0.78 for the negative dimension (32). In this study, the reliability coefficient of the scale was found to be 0.755.

### 2.5 Evaluation of data

Data analysis was performed using the SPSS 28 package program. The descriptive statistics of the qualitative variables in the study were given as numbers and percentages, and the descriptive statistics of the quantitative variables were given as mean, median, standard deviation, minimum and maximum. The conformity of quantitative variables to normal distribution was examined using the Shapiro–Wilk test. For non-normally distributed variables, the Mann–Whitney U test was used to compare the mean of two independent groups. The Kruskal-Wallis test was used for the mean comparison of more than two independent groups. The reliability of the scales was evaluated with the Cronbach alpha coefficient. A *p* < 0.05 value was accepted for statistical significance.

## 3 Results

The findings regarding the sociodemographic characteristics of the individuals constituting the sample of this study are presented in Table 1. Accordingly, the mean age of the participants was 29.71 ± 4.75 years, 39.8% were high school graduates, 71.8% were not employed, 14.6% had a chronic disease, 40% were between the 6th and 9th month of pregnancy, and 46.8% were between the 6th and 9th month of the postpartum period. It was also determined that 72.8% of the participants were non-smokers; the mean number of pregnancies was 2.12 ± 1.21 and the mean number of children was 1.27 ± 0.92.

**Table 1:** Socio-demographic characteristics of individuals (*n* = 103).

	*n*	%
Educational status		
Primary school	27	26.2
High school	41	39.8
Associate Degree	13	12.6
Undergraduate and Postgraduate	22	21.4
Working status		
Not working	74	71.8
Private sector	20	19.4
Public	9	8.7
Chronic disease		
Yes	15	14.6
No	88	85.4
Pregnancy month		
First 3 months	16	27.1
3–6 Months	19	32.2
6–9 Months	24	40.7
Breastfeeding period		
First 3 months	7	14.9
3–6 Months	18	38.3
6–9 Months	22	46.8
Cigarette		
I do not use	75	72.8
Less than one pack a day	13	12.6
1 Pack a day	11	10.7
One pack per week	4	3.9
Continuous variables Mean ± Sd Med (Min-Max)		
Age	29.7 ± 4.75	30(19–42)
The average number of pregnancies	2.12 ± 1.21	2(1–7)
Average number of children	1.27 ± 0.92	1(0–4)

The findings of the individuals included in the study regarding COVID-19 are presented in Table 2. Accordingly, it was determined that 76.6% of the participants had mild COVID-19, 27.3% had family members with severe disease, and 37.9% had the disease during breastfeeding. It was also determined that 74.8% of the participants received COVID-19 vaccine, 52% received at least 2 doses of vaccine, 78.4% received vaccine in the pre-pregnancy period, 74% preferred BioNTech vaccine, and 18.4% received flu vaccine. Among the factors affecting attitudes toward COVID-19 vaccination, the top two statements were ‘COVID-19 is a very dangerous disease’ (41.2%) and ‘Vaccines are recommended by the Ministry of Health’ (38.2%). The top two reasons for not getting COVID-19 vaccine were ‘I have reservations due to side effects’ (16.7%) and ‘I do not trust the protection of the vaccine’ (11.8%).

**Table 2:** Findings of individuals for COVID-19 (*n* = 103).

	*n*	%
Level of transmission of COVID-19		
Light	32	31.1
Heavy	33	32.0
I did not have the disease	38	36.9
Severe family history of COVID-19		
Yes	28	27.3
No	75	72.8
Period of contracting the COVID-19		
Pre-pregnancy	37	35.9
Pregnancy	27	26.2
Breastfeeding period	39	37.9
COVID-19 vaccination status		
Yes	77	74.8
No	26	25.2
COVID-19 vaccination period		
Pre-pregnancy	58	78.4
Pregnancy and beyond	16	21.6
COVID-19 vaccine type		
Sinovac	17	22.1
BioNTech	57	74
Turkovac	3	3.9
Flu vaccination status		
Yes	19	18.4
No	84	81.6
Factors affecting attitude toward COVID-19 vaccine		
COVID-19 is a very dangerous disease	42	41.2
Recommendation of vaccines by the Ministry of Health	39	38.2
No other option to protect from COVID-19	31	30.4
Social environment	15	14.7
Reason for not getting a COVID-19 Vaccine		
I have reservations because of the side effect	17	16.7
I do not trust the protection of the vaccine	12	11.8
I think there are not enough scientific studies done	11	10.8
I think that I can strengthen my immunity with herbal and supportive applications instead of vaccination.	8	7.8

The total and sub-dimension mean scores of the Vaccine Hesitation Scale in Pandemics (VHSP) are presented in Table 3. Accordingly, it was determined that the participants scored 30.83 ± 6.91 points from the scale with a maximum total score of 50. It was determined that the mean score of the ‘Lack of Confidence’ sub-dimension of the VHSP was 25.07 ± 6.84 and the mean score of the ‘Risk’ sub-dimension was 5.77 ± 1.53.

**Table 3:** Mean, standard deviation, median, minimum, maximum, and Cronbach alpha values of the scale and its sub-dimensions (*n* = 103).

α		Mean ± Sd Med(Min-Maks)
Vaccine hesitancy scale in pandemics (VHSP)	
Lack of Confidence	25.07 ± 6.84 26(8.00–40.00)
Risks	5.77 ± 1.53 6(2.00–10.00)
Total 0.831	30.83 ± 6.91 32(11.00–50.00)
Attitudes toward COVID-19 vaccine scale (ATCVS)	
Positive Attitude	13.16 ± 4.31 14(4.00–20.00)
Negative Attitude	17.63 ± 3.62 18(9.00–25.00)
Total 0.755	25.50 ± 5.20 26(9.00–35.00)

α, Cronbach Alfa.

The total and sub-dimension mean scores of the Attitudes toward COVID-19 Vaccine Scale (ATCVS) are also shown in Table 3. It was determined that the participants scored 25.50 ± 5.20 points from the scale with a maximum total score of 45. The mean score of the ‘Positive Attitude’ sub-dimension of the ATCVS was 13.16 ± 4.31 and the mean score of the ‘Negative Attitude’ sub-dimension was 17.63 ± 3.62.

Some demographic variables affecting the level of hesitation and attitude of the participants toward COVID-19 vaccine are given in Table 4. When the demographic variables affecting the total score of the VHSP were analyzed, it was found that there was a significant difference in terms of the variable ‘working status’ (*p* = 0.015). When the differences were examined in detail, it was found that the mean VHSP total score of the participants working in the private sector was significantly higher than the participants ‘not working’ in any institution and ‘working in a public institution’ (*p* = 0.034; *p* = 0.006). There was also a significant difference between the mean total score of the VHSP and the variables of ‘flu vaccination’ (*p* = 0.016). When the differences were examined in detail, it was determined that the mean total score of the VHSP was higher in participants who did not have influenza vaccination.

**Table 4:** Some demographic variables that affect the level of hesitation and attitude toward the COVID-19 vaccine (*n* = 103).

Variables	Vaccine hesitancy scale in pandemics (VHSP)						Attitudes toward COVID-19 vaccine scale (ATCVS)					
	Lack of confidence		Risks		Total		Positive attitude		Negative attitude		Total	
	Mean ± Sd		Med (Min-Max)	Mean ± Sd		Med (Min-Max)	Mean ± Sd		Med (Min-Max)	Mean ± Sd		Med (Min-Max)	Mean ± Sd		Med (Min-Max)	Mean ± Sd		Med (Min-Max)	*p*	*p*	*p*	*p*	*p*	*p*
Working status												
Not working	24.28 ± 7.05	24.50(8–37)	5.76 ± 1.54	6(2–9)	30.04 ± 6.90	31(11–43)	12.81 ± 4.08	13(4–20)	17.25 ± 3.53	17(9–25)	25.52 ± 5.07	27(9–35)
Private sector	28.70 ± 5.23	29.50(19–40)	6.10 ± 1.51	6(2–10)	34.80 ± 5.47	35.5 (25–50)	14.75 ± 4.54	16(6–20)	18.65 ± 3.63	19(11–25)	26.10 ± 3.47	26(19–33)
Public	23.44 ± 6.08	26(14–30)	5.11 ± 1.45	5(2–7)	28.55 ± 7.26	31(18–36)	12.44 ± 5.29	13(4–19)	18.44 ± 4.18	19(13–25)	24.00 ± 8.84	28(9–33)
	*p* = 0.025*		*p* = 0.244		*p* = 0.015*		*p* = 0.094		*p* = 0.227		*p* = 0.980	
Chronic disease												
Yes	24.87 ± 8.07	28(8–36)	5.20 ± 1.89	6(2–8)	30.06 ± 8.48	34(12–38)	12.40 ± 5.69	13(4–20)	19.47 ± 3.79	19(11–25)	22.93 ± 7.29	24(9–32)
No	25.1 ± 6.66	25(8–40)	5.86 ± 1.45	6(2–10)	30.96 ± 6.66	31.5(11–50)	19.47 ± 3.79	14(4–20)	17.31 ± 3.51	17(9–25)	25.94 ± 4.67	27(9–35)
	*p* = 0.725		*p* = 0.299		*p* = 0.757		*p* = 0.645		*p* = 0.021*		*p* = 0.157	
Smoking												
I do not use	24.39 ± 7.10	25(8–37)	5.60 ± 1.58	6(2–9)	29.98 ± 6.99	31(11–43)	12.81 ± 4.37	13(4–20)	17.93 ± 3.70	18(10–25)	24.85 ± 4.88	25(9–33)
1 pack of six per day	26.00 ± 7.18	25(8–40)	6.31 ± 1.37	6(4–10)	32.30 ± 8.30	32(12–50)	13.54 ± 4.94	14(4–20)	17.00 ± 4.16	17(9–25)	26.53 ± 7.72	28(9–35)
1 pack a day	28.36 ± 3.85	30(20–34)	6.00 ± 1.48	6(4–8)	34.36 ± 3.00	35(28–38)	15.27 ± 2.83	16(8–20)	16.18 ± 2.82	17(11–20)	29.09 ± 2.38	25(27–33)
1 pack per week	25.75 ± 6.13	26.5(18–32)	6.50 ± 0.57	6.5(6–7)	32.25 ± 6.07	32.5(25–39)	12.50 ± 4.04	13.5(7–16)	18.00 ± 0.81	18(17–19)	24.50 ± 3.41	25(20–28)
	*p* = 0.198		*p* = 0.284		*p* = 0.132		*p* = 0.230		*p* = 0.432		*p* = 0,004*	
COVID-19 vaccination period												
Pre-pregnancy	25.97 ± 7.54	28(8–40)	5.72 ± 1.44	6(2–10)	31.68 ± 7.77	34(11–50)	13.97 ± 4.31	15(4–20)	18.21 ± 3.27	18(11–25)	25.75 ± 5.29	26.5(11–33)
Pregnancy and beyond	26.81 ± 6.26	27.5(17–36)	4.88 ± 1.31	5(2–6)	31.68 ± 6.40	33(19–40)	14.44 ± 4.33	16(4–20)	19.27 ± 2.89	19(14–25)	25.12 ± 5.38	27(9–30)
	*p* = 0.838		*p* = 0.014*		*p* = 0.875		*p* = 0.340		*p* = 0.165		*p* = 0.787	
Getting a flu shot												
Yes	22.21 ± 6.90	22(8–36)	5.32 ± 1.60	6(2–7)	27.52 ± 7.49	29(11–40)	11.05 ± 4.62	12(4–18)	18.37 ± 3.89	18(9–25)	22.68 ± 6.70	22(11–35)
No	25.71 ± 6.70	26.5(8–40)	5.87 ± 1.51	6(2–10)	31.58 ± 6.59	33(14–50)	13.63 ± 4.12	15(4–20)	17.46 ± 3.56	17(10–25)	26.14 ± 27.0	27(9–33)
	*p* = 0.023*		*p* = 0.291		*p* = 0.016*		*p* = 0.035*		*p* = 0.302		*p* = 0.019*	

*p* < 0.05.

It was concluded that there was a significant difference in the mean score of the ‘Lack of Confidence’ sub-dimension of the VHSP scale in terms of ‘working status’ (*p* = 0.025). When the differences were examined in detail, it was found that the mean score of the ‘Lack of Confidence’ sub-dimension of those working in the private sector was significantly higher than the participants ‘not working’ in any institution and ‘working in a public institution’ (*p* = 0.043; *p* = 0.010). It was also determined that there was a significant difference in terms of the variables of ‘having flu vaccination’ (*p* = 0.023) according to the mean score of the ‘Lack of Confidence’ sub-dimension of the VHSP scale. Accordingly, it was determined that the mean score of the ‘Lack of Confidence’ sub-dimension of the participants who did not have flu vaccination was higher than those who had flu vaccination (*p* = 0.023).

According to the mean score of the ‘Risk’ sub-dimension of the VHSP scale, it was determined that there was a significant difference in terms of the variable ‘vaccination period’ (*p* = 0.014). Accordingly, it was determined that the mean scores of those who were vaccinated in the pre-pregnancy period were higher.

When the participants’ ATCVS total scores were analyzed, it was determined that there was a significant difference in terms of ‘smoking status’ (*p* = 0.004). Accordingly, it was determined that the mean ATCVS total scores of non-smokers were significantly lower than those who smoked ‘less than one pack a day’ and ‘more than one pack a day’. The mean ATCVS total scores of participants who smoked ‘more than one pack a day’ were significantly higher than those who smoked ‘more than one pack a week’. It was also determined that there was a significant difference between the participants’ ATCVS total scores and the variable of ‘flu vaccination’ (*p* = 0.019). Accordingly, it was determined that the mean total score of the ATCVS of those who did not have influenza vaccination was significantly higher than those who had influenza vaccination.

According to the ‘Positive Attitude’ sub-dimension of the ATCVS, it was determined that there was a significant difference in terms of the variable ‘having flu vaccination’ (*p* = 0.035). Accordingly, it was determined that the mean scores of the ‘Positive Attitude’ sub-dimension of those who did not receive flu vaccination were higher.

According to the ‘Negative Attitude’ sub-dimension of the ATCVS, a significant difference was found in terms of ‘chronic disease’ (*p* = 0.021). It was concluded that the ‘Negative Attitude’ mean scores of those with chronic diseases were higher.

No significant difference was found between the mean total score and sub-dimensions of the VHSP and ATCVS scales in terms of the variables ‘age’, ‘educational status’, ‘number of pregnancies and children’, ‘number of months of pregnancy or postpartum period’, ‘presence of infants, children and individuals over 65 years of age among the family members living together’ (*p* > 0.05).

Table 5 shows the comparison of the participants’ VHSP and ATCVS scale sub-dimension and total mean scores. Accordingly, it was determined that there was a significant positive correlation between the total VHSP score and the total ATCVS score (*r* = 0.350; *p* < 0.001). It was determined that there was a significant positive correlation between the mean total score of the VHSP and the mean scores of the ‘Positive Attitude’ (*r* = 0.538; *p* < 0.001) and ‘Lack of Confidence’ (*r* = 0.970; *p* < 0.001) sub-dimensions. There was a significant positive correlation between the mean total score of ATCVS and the mean scores of ‘Positive Attitude’ (*r* = 0.652; *p* < 0.001), ‘Negative Attitude’ (*r* = 0.502; *p* < 0.001) and ‘Lack of Confidence’ (*r* = 0.352; *p* < 0.001) sub-dimensions. It was also determined that there was a significant positive correlation between the mean scores of ‘Lack of Confidence’, which is a sub-dimension of VHSP, and ‘Positive Attitude’, which is a sub-dimension of ATCVS (*r* = 0.652; *p* < 0.001).

**Table 5:** Comparison of VHSP and ATCVS scale sub-dimensions and total score averages (*n* = 103).

		Negative attitude	Lack of confidence	ATCVS total	VHSP total
Positive attitude	**r**	−0.232	0.602	0.652	0.538
	**p**	0.018	<0.001*	<0.001*	<0.001*
	n	103	103	103	103
Negative attitude	r		−0.134	0.502	−0.050
	p		0.176	<0.001*	0.615
	n		103	103	103
Lack of confidence	r			0.352	0.970
	p			<0.001*	<0.001*
	n			103	103
ATCVS total	r				0.350
	p				<0.001*
	n				103

*p < 0.05.

## 4 Discussion

In this study, which was conducted to determine the factors associated with the hesitation and attitude levels of individuals during pregnancy and breastfeeding toward COVID-19 vaccines, the fact that the total and sub-dimension scores of the VHSP are above the average score indicates that the participants have a high level of hesitation toward the COVID-19 vaccine. Lack of confidence in the vaccine and high-risk perception were also effective in increasing the level of hesitation. In a multinational cross-sectional study conducted with 16 thousand participants, it was found that 40–50% of the participants were hesitant about the COVID-19 vaccine. It was also reported that this rate was higher in pregnant individuals compared to breastfeeding individuals (33). In a China-based cross-sectional study, the acceptance status of COVID-19 vaccines by pregnant women and related factors were examined in the context of the health belief model, and it was reported that approximately one-quarter of the participants were hesitant about vaccination (30). Individuals who are generally hesitant about vaccination are skeptical about new vaccines or have varying degrees of ambivalence about vaccines. Shih et al. argued that general vaccine hesitancy is strongly associated with the rejection of COVID-19 vaccines (22).

Vaccine hesitancy is commonly due to a lack of confidence and knowledge about vaccine safety and efficacy. Widespread concerns about the safety of the vaccine, a reason for hesitancy toward COVID-19 vaccines, are associated with individuals not wanting to jeopardize their health against the uncertainties of a newly produced vaccine. Safety concerns include the lack of accurate and safe data on the vaccine, the speed of vaccine development, the lack of knowledge about the short and long-term side effects of the vaccine, and trust in the healthcare system (20).

Although safety concerns regarding vaccine hesitancy were at the forefront in this study, as in the literature, in a study examining the safety and efficacy of COVID-19 vaccines, it was concluded that mRNA vaccine did not increase pregnancy complication rates and reduced the risk of COVID-19 infection up to five times (20). Side effects such as injection site pain, numbness, redness, swelling, fever, headache, as well as neurological, psychological and cardiac complications that develop as a result of COVID-19 vaccines negatively affect the level of confidence in the vaccine. Research results show that the risk of side effects is high, especially in individuals who prefer to live vaccines, are over 50 years old, male, smoke, live in urban areas, and have underlying diseases (34–37).

A UK-based study examining the attitudes of pregnant women toward vaccination revealed that the risk posed by COVID-19 vaccines was perceived as more dangerous than the risks arising from COVID-19 (38). While promoting protective behaviors for COVID-19, it is critical to understand how the risk posed by the disease is perceived. As stated in the health belief model, the tendency to take preventive measures increases when the risk of disease is perceived as high (8). The findings of this study suggest that the participants’ perceived risk of COVID-19 vaccines is higher than the perceived risk of COVID-19. In the emergence of these results, which are also supported by the findings in the literature, it is thought that the collection of research data during the period, when there were many unknowns about vaccines and their effects, public debates on both safety and necessity of vaccination continued.

In this study, the fact that ATCVS total and sub-dimension scores are above the average score indicates that the participants have developed a positive attitude toward the COVID-19 vaccine and have built awareness about the vaccine. In studies conducted with different sample groups, unlike the results of this study, it was determined that participants commonly had negative attitudes toward COVID-19 vaccines (7, 15). In a study examining women’s attitudes toward COVID-19 vaccination, it was reported that pregnant individuals were more likely to refuse the vaccine than non-pregnant and breastfeeding individuals (39). The main reason for negative attitudes toward COVID-19 vaccines is lack of knowledge and trust. It is thought that adequate and appropriate education on vaccination will positively affect attitudes. According to the result of this study, which is also supported by the literature findings, the participants’ acceptance of vaccination, despite hesitation, indecision and insecurity caused by the unknowns about COVID-19 vaccines, is associated with the fact that they do not want to risk the health of their babies nor themselves (30). In addition, it is thought that the dissemination of content on the benefits of vaccines in the visual and written media and the guidance of health professionals are effective in the emergence of this result.

In this study, the total and sub-dimensional mean scores of VHSP and ATCVS were compared and it was determined that there was a significant positive relationship. It was found that there was a significant positive correlation between the mean total score of VHSP and the mean scores of ‘Lack of Confidence’ and ‘Positive Attitude’ sub-dimensions, and between the mean total score of ATCVS and the mean scores of ‘Positive Attitude’, ‘Negative Attitude’ and ‘Lack of Confidence’ sub-dimensions. In addition, it was determined that there was a significant positive correlation between the mean scores of ‘Lack of Confidence’, which is a sub-dimension of VHSP, and ‘Positive Attitude’, which is a sub-dimension of ATCVS. This result indicates that the level of indecision increased with the increase in the level of insecurity of the participants toward the COVID-19 vaccine, but despite this, they accepted the vaccination by exhibiting a positive attitude.

In the study by Harada et al., anxiety and risk perception toward COVID-19 were found to be significantly associated with vaccine acceptance, supporting the results of this study (20). In the study by Sıhıh et al., a significant positive correlation was found between COVID-19 vaccine hesitancy and vaccine refusal (22). In a study examining the factors affecting the attitude toward COVID-19 vaccines, it was found that there was a moderate and significant relationship between positive attitude and trust variables, supporting the results of this study (9). In another study, it was concluded that although the vaccination rates of the participants were high, they had serious hesitations about the COVID-19 vaccine. It was also reported that there was a positive correlation between COVID-19 anxiety and vaccine hesitancy (40). The fact that pregnant and breastfeeding individuals accept vaccination despite hesitation, indecision and insecurity caused by the unknowns about COVID-19 vaccines is associated with the fact that the perceived risk of COVID-19 is higher than the perceived risk of side effects of the vaccine.

Approximately 70% of the population needs to be vaccinated for the COVID-19 vaccine to create social immunity. The data of this study were collected during the period when public debate on the safety and necessity of vaccination against COVID-19 was ongoing. Therefore, it is expected and supported by the findings in the literature that pregnant and breastfeeding individuals, especially in the disadvantaged group against infectious diseases, accept vaccination for various reasons such as protecting the health of themselves and those around them, especially the health of their babies, despite the high level of hesitation toward vaccination (9, 38). These results suggest that even during a pandemic, when attitudes toward vaccines are critical, hesitations about the safety of vaccines can be as influential as perceived disease risks. It is also supported that individuals who are hesitant about general vaccines are also hesitant about COVID-19 vaccines (41). In line with these results, it is thought that informing the community about COVID-19 and vaccines by all healthcare professionals, especially nurses, who assume the role of educators in health care practices, will reduce the level of fear and anxiety and thus increase vaccination rates.

In this study, some demographic variables affecting the level of hesitation and attitude of the participants toward the COVID-19 vaccine were examined (Table 4). Accordingly, the level of hesitation and distrust toward COVID-19 vaccines was found to be significantly higher among those working in the private sector. While the study by Wagner et al. supported this result, Ceulemans et al. reported that participants who were not working in any job had a high level of hesitation toward COVID-19 vaccines (33, 41). As the risk of exposure to COVID-19 will increase due to long working hours and increased workload in the private sector, the level of hesitancy of individuals toward COVID-19 vaccines would be expected to be low. This result suggests that other protective measures to prevent transmission may have been preferred more by private sector employees.

In this study, it was found that those who were vaccinated in the pre-pregnancy period had high risk perceptions toward COVID-19 vaccine. In the meta-analysis study by Shamshirsaz et al., it was stated that vaccination rates during pregnancy were low, although there was insufficient evidence of the mortality and morbidity risk of COVID-19 vaccine on mother and baby (7). It is thought that the high perceived risk of COVID-19 before pregnancy may have led individuals to practice protective behaviors such as vaccination. Participants who perceive the risk for the disease at a low level may have wanted to see the effects of the vaccine on other individuals in line with the “wait and see” approach before being vaccinated (8, 41).

According to this study, it was determined that participants who did not have a flu vaccine had a higher level of hesitation and distrust toward COVID-19 vaccines, but had a positive attitude toward COVID-19 vaccines. In the literature, there are studies that indicate the level of hesitation and distrust toward COVID-19 vaccines among those who have not been vaccinated against influenza was high. This result is consistent with the knowledge that individuals with prejudice toward vaccination also approach COVID-19 vaccines with caution (42, 43). It is thought that the participants’ thinking that the measures taken for the COVID-19 will reduce exposure to the flu virus may have been effective in the emergence of this result. There are studies in the literature that support this result, as well as studies that concluded that individuals who did not receive influenza vaccination were less likely to accept COVID-19 vaccines (38, 42–44). In a meta-analysis study, it was reported that those who received influenza and TDAP vaccines during pregnancy also had high rates of acceptance of COVID-19 vaccines (7). The results from this study are consistent with the data on vaccine hesitancy, which has been a growing problem in public health over the past decade (15, 29).

In this study, it was determined that individuals with chronic diseases had more positive attitudes toward COVID-19 vaccines. It is thought that the tendency of individuals to preventive behaviors may be effective in the emergence of this situation due to the poor prognosis of COVID-19 in individuals with chronic diseases. In an Italy-based study, it was reported that most of the participants’ cohabitants or individuals in their close environment were in high disease risk categories and this positively affected the intention to be vaccinated (44). In contrast to these data, in studies planned with individuals with different chronic diseases, it was concluded that vaccination intention was lower compared to the general population (8, 9). There are also studies reporting that individuals with good general health are less likely to accept the COVID-19 vaccine (43).

In this study, it was concluded that variables such as: ‘age’, ‘educational status’, ‘number of pregnancies and children’, ‘number of months of pregnancy or postpartum period’, ‘presence of infants, children and individuals over 65 years of age among family members living together’ did not significantly affect the level of hesitation and attitude toward vaccination (*p* > 0.05). Contrary to the results of this study, there are studies which conclude that increasing age, education level, occupation, period and number of pregnancies positively affect attitudes toward COVID-19 vaccination and increase the acceptance of these vaccines. It has been emphasized that vaccine acceptance will increase as vulnerability to environmental factors will increase with age (5, 8, 19, 28, 33). In addition, healthcare workers, who are reliable sources of information about vaccination, have a decisive role in influencing the vaccination decisions of the patients they come into contact with. Therefore, it has been stated that informing healthcare workers correctly about the vaccine and ensuring their vaccination is effective in controlling the pandemic can be of great importance. Especially nurses who are in one-to-one contact with patients should organize health trainings by their roles as educators and counselors to protect public health (45–47). The results obtained show that attitudes and hesitation toward COVID-19 vaccines may vary according to various situational factors. It is recommended that these factors be taken into account in the trainings to be planned.

Last but not least, since the research data was collected during a period of uncertainty about the effects of the pandemic and vaccines, the results obtained can be considered as the immediate reactions of the participants. For effective immunization studies, it is important to re-evaluate the factors affecting the level of hesitation toward vaccination under changing conditions. In this context, it is recommended to plan new studies with more sample groups and multiple follow-ups.

## 5 Conclusion

According to the findings from this study, it was concluded that although the level of hesitation and attitude toward COVID-19 vaccines was high in the sample consisting of individuals during pregnancy and breastfeeding, they exhibited a positive attitude. It was determined that the level of hesitation and attitude of the participants toward vaccines was affected by various situational variables. Accordingly, it was determined that the level of hesitation toward the COVID-19 vaccine was associated with the variables ‘Working status’, ‘Flu vaccination status’ and ‘Period of vaccination’. It was determined that the level of attitude toward COVID-19 vaccine was associated with the variables ‘Smoking’, ‘Flu vaccination status’ and ‘Chronic disease’. It is seen that the level of perceived risk, insecurity and ambivalence toward COVID-19 vaccines is not at a level to prevent the acceptability of COVID-19 vaccine.

To increase vaccine acceptance, it is important to understand individuals’ concerns and perceptions about vaccination. Considering the changing nature of COVID-19 and uncertainties regarding vaccine development for resistant variants, it is suggested that studies determine the level of vaccine hesitancy, attitudes and associated factors be re-evaluated at regular intervals with a larger sample group. In addition, taking into account the anti-scientific attitudes of individuals with high levels of vaccine hesitancy, it is suggested that researchers provide trainings with content appropriate to their sociodemographic characteristics. Therefore, it is essential to ensure interdisciplinary cooperation in the fight against vaccine hesitancy, which has a complex and multifactorial structure.

### 5.1 Limitations

Research data were collected during the period when COVID-19 continued to be seen in Turkey.

During the pandemic period, there were difficulties in reaching more participants due to the low number of patients applying to health institutions due to the risk of transmission and individuals’ concerns and prejudices about participating in research.

Individuals who were invited to participate in the study generally stated that they did not want to participate in the study because they wanted to leave the hospital environment as soon as possible due to the risk of infection. Therefore, it was difficult to reach a larger number of patients.

During the planning phase of the study, care was taken to comply with the deadline specified to the ethics committee, so the data collection period could not be extended to increase the sample size.

Findings from the study should not be generalized to the whole population due to limited sample representation.

## Data availability statement

The raw data supporting the conclusions of this article will be made available by the authors, without undue reservation.

## Ethics statement

The studies involving humans were approved by University of Health Sciences Hamidiye Scientific Research Ethics Committee 11.03.2022 (22/136). The studies were conducted in accordance with the local legislation and institutional requirements. The participants provided their written informed consent to participate in this study. Written informed consent was obtained from the individual(s) for the publication of any potentially identifiable images or data included in this article.

## Author contributions

NS: Conceptualization, Data curation, Formal analysis, Investigation, Methodology, Project administration, Resources, Software, Supervision, Validation, Visualization, Writing – original draft, Writing – review & editing. EA: Conceptualization, Data curation, Investigation, Methodology, Project administration, Resources, Software, Supervision, Validation, Visualization, Writing – original draft, Writing – review & editing. RG: Conceptualization, Data curation, Investigation, Methodology, Project administration, Resources, Software, Supervision, Validation, Visualization, Writing – original draft, Writing – review & editing. OP: Conceptualization, Data curation, Formal analysis, Investigation, Methodology, Project administration, Resources, Software, Supervision, Validation, Visualization, Writing – original draft, Writing – review & editing.
